# Classification of small ruminant lentivirus subtype A2, subgroups 1 and 2 based on whole genome comparisons and complex recombination patterns

**DOI:** 10.12688/f1000research.27898.1

**Published:** 2020-12-11

**Authors:** Aaron M. Dickey, Timothy P. L. Smith, Michael L. Clawson, Michael P. Heaton, Aspen M. Workman

**Affiliations:** 1 US Department of Agriculture, Agricultural Research Service, US Meat Animal Research Center, Clay Center, NE, 68933, USA

**Keywords:** Small ruminant lentivirus, recombination, quasispecies, ovine progressive pneumonia virus

## Abstract

**Background:** Small ruminant lentiviruses (SRLVs) cause a multisystemic chronic wasting disease in sheep across much of the world. SRLV subtype A2 is prevalent in North America and further classified into multiple subgroups based on variation in the group antigens gene ( *gag*) and envelope ( *env*) genes. In sheep, the ovine transmembrane protein 154 ( *TMEM15*4) gene is associated with SRLV susceptibility. Ewes with at least one copy of *TMEM154* encoding a full-length protein with glutamate at position 35 (E35; haplotypes 2 and 3), are highly susceptible to SRLV infection while ewes with any combination of *TMEM154* haplotypes which encodes lysine (K35; haplotype 1), or truncated proteins (haplotypes 4 and 6) are several times less so. A2 subgroups 1 and 2 are associated with host *TMEM154* genotypes; subgroup 1 with the K35/K35 genotype and subgroup 2 with the E35/E35 genotype.

**Methods: **The goals of this study were to analyze sequence variation within and among SRLV subtype A2 subgroups 1 and 2 and to identify genome-scale recombination patterns. This was done using full-length assemblies of virus samples.

**Results:** Consensus viral genomes were assembled for 23 infected sheep, including animals of assorted *TMEM154 *genotypes comprised of haplotypes 1, 2, or 3. Viral genome analysis identified viral subgroups 1 and 2 among the samples, and revealed additional sub-structure within subgroup 2 based on models predicting complex patterns of recombination between the two subgroups in several genomes. Animals with evidence of dual subgroup infection also possessed the most diverse quasi-species and the most highly recombined genomes.

**Conclusions:** The viral subgroup framework developed to classify SRLV consensus genomes along a continuum of recombination suggests that animals with the *TMEM154* E35/K35 genotype may represent a reservoir for producing viral genomes representing recombination between A2 subgroups 1 and 2.

## Introduction

Small ruminant lentiviruses (SRLV) are a genetically diverse group of lentiviruses belonging to the family *Retroviridae*. SRLV infect sheep, goats, and wild ruminants worldwide causing a lifelong persistent infection clinically characterized by wasting, interstitial pneumonia with labored breathing, indurative mastitis, arthritis, and/or more rarely, encephalitis ( Blacklaws, 2012). Disease progression is typically slow, and the genetic background of both the host and virus influence the clinical course and outcome of infection ( Heaton *et al.,* 2012; Sider *et al.,* 2013).

The SRLV genome consists of two identical positive-sense single-stranded RNA subunits approximately 9 kb in length. The viral genome, which is integrated into host cells in the form of a provirus, contains three structural genes ( *gag*, *pol*, and *env*) and three regulatory genes ( *tat*, *vif,* and *rev*) flanked by non-coding long terminal repeat regions (LTRs). Phylogenetic analysis based on partial group antigens gene ( *gag*) and polymerase ( *pol)* gene sequences divides these viruses into five major genotype groups, A-E, which are further divided into different subtypes ( Shah *et al.,* 2004). The groups differ by 25–37% and the subtypes differ by 15–27% in nucleotide composition at these genomic loci ( Ramírez *et al.,* 2013). Genotypes A and B are distributed worldwide, while genotypes C-E are geographically restricted ( Gjerset *et al.,* 2006; Grego *et al.,* 2009; Shah *et al.,* 2004).

The comprehensive set of haplotypes from the same viral species in a single host is known as a quasispecies ( Eigen *et al.,* 1988). The viral quasispecies arises from the interplay of three evolutionary factors throughout the duration of a chronic infection. These three factors are mutation, recombination, and selection. Point mutations and small indels are introduced into the SRLV viral genome due to its low fidelity, error prone reverse transcriptase enzyme. Selection can be driven by the host immune system and antiviral medications. Work with related lentiviruses has revealed selection pressure on mutants can produce variants diverging by up to 5% from the founder strain in a few years, though this rate does not remain constant over time ( Lee *et al.,* 2008; Shankarappa *et al.,* 1999). Co-infection (simultaneous infection) or superinfection (sequential infection) can lead to more dramatic genetic changes through recombination, but these two types of dual infection are difficult to distinguish in the absence of a detailed infection history. Regardless of the timing, when diverse viral subtypes infect the same host cell, the reverse transcriptase readily switches between viral genomes, with estimates of three to nine recombination events per replication cycle ( Jetzt *et al.,* 2000). This process allows rapid emergence of new viral strains that may exhibit novel phenotypes (reviewed in Ramírez *et al.,* 2013). *In vivo* recombination has been documented between genotypes A and B and among genotype A and B subtypes ( Andrésdóttir, 2003; Fras *et al.,* 2013; Pisoni *et al.,* 2007; Ramírez *et al.,* 2011; Sider *et al.,* 2013). However, recombination in SRLVs has not previously been characterized at the whole-genome level.

The virus-host interaction is a continuous co-evolutionary process. In sheep, genetic variation in the host transmembrane protein 154 ( *TMEM154*) gene associates with SRLV susceptibility ( Heaton *et al.,* 2012; Leymaster *et al.,* 2013; Leymaster *et al.,* 2015; Molaee *et al.,* 2018; Molaee *et al.,* 2019; Yaman *et al.,* 2019). Twelve *TMEM154* haplotypes have been identified ( Heaton *et al.,* 2012), and ewes homozygous for haplotype 1, which encodes a lysine at position 35 (K35), had a decreased risk of SRLV infection compared to those with one or two copies of haplotype 2 or 3 (ancestral), both of which encode a glutamate at position 35 (E35). Two distinct SLRV A2 subgroups have been identified that infected sheep in association with their *TMEM154* E35K genotypes and specific diplotypes ( Sider *et al.,* 2013). SLRV A2 subgroup 1 viruses were significantly more likely to infect sheep with either *TMEM154* diplotypes 1,1 or 1,4. *TMEM154* haplotype 4 contains a rare frameshift mutation (A4Δ53) and does not produce mRNA encoding functional amino acids downstream of amino acid position 4 of the precursor protein. Consequently, subgroup 1 associated with hemizygous or homozygous K35 genotypes. SLRV A2 subgroup 2 viruses were more likely to infect sheep with one or two copies of either haplotype 2 or 3, and that could also have one copy of haplotype 4 ( Sider *et al.,* 2013). Consequently, subgroup 2 viruses associated with hemizygous or homozygous E35 genotypes. While it has been proposed that *TMEM154* E35K hemizygosity or homozygosity could be a factor in SRLV A2 subgroup associations, the biology remains obscured.

SRLV A2 subgroups 1 and 2, and their associations with *TMEM154* E35K are not well understood. The subgroups were previously defined by sequence variation in a partial region of the *gag* and the transmembrane region of the envelope gene ( *env*), which were not thought to have critical roles in potential SRLV *TMEM154* interactions ( Sider *et al.,* 2013). Due to relatively short sequenced regions of SRLV A2 subgroups 1 and 2 genomes and because of extensive recombination detected within the sequences, the cutoff between groups 1 and 2 was not clear ( Sider *et al.,* 2013). In this study, we obtained full-length consensus SRLV genomes from a cross-section of sheep belonging to the flock which was part of the original *TMEM154* association studies ( Heaton *et al.,* 2012; Heaton *et al.,* 2013; Leymaster *et al.,* 2013; Leymaster *et al.,* 2015; Sider *et al.,* 2013). These sheep had all been genotyped as containing haplotypes 1, 2, or 3. Ovine *TMEM154* haplotypes 4-12 were not represented in this study. The goals of this study were to 1) obtain full-length consensus genomes for members of SLRV A2 subgroups 1 and 2; 2) identify subgroup 1 and 2 specific variants, while accounting for recombination, and use these variants to estimate levels of intra-host sequence variation; 3) search for subgroup-specific functional viral variants relative to host *TMEM154* E35K genotypes.

## Methods

### Ethics statement

All animal procedures were reviewed and approved by the United States Department of Agriculture (USDA), Agricultural Research Service (ARS), U.S. Meat Animal Research Center (USMARC) Animal Care and Use Committee prior to their implementation (Experiment Number 96). The source flock’s history of disease surveillance is also relevant when requesting reference samples described in any report. Since first stocking sheep in 1966, USMAC has not had a known case of scrapie. Until 2002, surveillance consisted of monitoring sheep for possible signs of scrapie and submitting brain samples to the USDA Animal and Plant Health Inspection Service (APHIS) National Veterinary Services Laboratory in Ames, IA for testing. All tests have been negative. Since April 2002, USMARC has voluntarily participated in the APHIS Scrapie Flock Certification Program, is in compliance with the National Scrapie Eradication Program, and is certified as scrapie-free. With regards to other transmissible diseases, it is recognized that the USMARC flock of 2000 to 4000 breeding ewes is located in a bluetongue medium incidence area and is known to have some prevalence of contagious ecthyma (sore mouth), foot rot, paratuberculosis (Johne’s disease), ovine progressive pneumonia (visna-maedi), and pseudotuberculosis caseous lymphadenitis.

### Study population

Lungs from 22 sheep at the US Meat Animal Research Center in Nebraska that were a part of the original study for association of A2 subgroups 1 and 2 with *TMEM154* haplotypes ( Heaton *et al.,* 2012; Sider *et al.,* 2013) were used in this study ( Table 1). These sheep were all genotyped as containing haplotypes 1, 2, or 3. SRLV seropositive sheep were originally diagnosed with clinical ovine progressive pneumonia (OPP) by gross morphology and histopathology of both lung and mediastinal lymph node. In addition, colostrum from one seropositive ewe (201373037) showing no clinical signs of disease was used in this study ( Table 1).

**Table 1.** Sheep and virus information and for 23 SRLV A2 strains.

Animal ID / Viral Strain	GenBank Accession Number	Breed	*TMEM154* Diplotype	Mean Genome Coverage ± Standard Deviation	Submitted Genome Length	Subgroup ^**^
200303038	MT993897	MARCIII	1,2	489.0±426.1	9192	1
200303332	MT993898	MARCIII	1,1	52.3±20.3 ^*^	9171	1
200303013	MT993896 ^***^	MARCIII	1,1	920.0±373.0	9206	1
200103515	MT993899	MARC III	1,1	71.3±19.4 ^*^	9166	1
200050064	MT993900	ROMANOV- DORSET-SU	1,2	482.8±185.7	9202	r/d
200323455	MT993901	MARCIII	1,1	1287.9±345.8	9194	1
200103342	MT993902	MARCIII	1,1	351.6±124.9	9207	1
199835918	MT993903	RAMBOUILLET	1,2	1344.8±440.8	9192	1
200023230	MT993904	MARCIII	1,1	1636.7±1223.3	9193	1
201373037	MT993905	KATHADIN X RAMBOUILLET	1,1	76.3±31.0 ^*^	9166	1
200117502	MT993906	RAMBOUILLET	1,3	395.8±252.6	9185	r/d
200216049	MT993907	FINN	1,3	540.2±280.9	9200	r/d
200212120	MT993908	POLLED DORSET	1,2	927.8±611.7	9202	r/d
200312013	MT993909	POLLED DORSET	2,2	651.0±410.0	9191	r/d
200312088	MT993910	POLLED DORSET	1,2	279.3±195.5	9199	r/d
199906011	MT993911 ^***^	TEXEL	2,2	692.7±417.4	9189	2a
200106932	MT993912	TEXEL	2,3	1230.6±1086.1	9191	2a
200106929	MT993913	TEXEL	2,2	580.3±521.8	9201	2a
200016283	MT993914	FINN	1,2	2014.5±710.3	9206	2b
200335185	MT993915	RAMBOUILLET	1,2	171.8±74.7	9203	2b
200177363	MT993916	DORSET X ROMANOV	1,3	1801.5±1564.7	9215	2b
199916128	MT993917	FINN	1,1	227.6±67.3 ^*^	9172	2b
199916193	MT993918	FINN	1,1	2661.1±1329.2	9204	r/d

*No long reads: coverage includes only short reads, 10 to 26 nucleotides not fully resolved on 5' ends. **The population to which a consensus genome was assigned in the subgroup 1 vs 2a vs 2b recombination model ( Figure 3A). r/d: recombinant/dual infections. ***The short read + long read consensuses reported here are identical (200303013) and slightly different (199906011) from the long-read consensuses reported by Workman *et al.,* 2017 (KY358787 and KY358788 respectively). See results.

### Generation of full-length SRLV genomes

Lung samples were homogenized using a gentleMACS dissociator (Miltenyl Biotec; San Diego, CA) in minimal essential medium (Gibco, Thermo Fisher Scientific, Waltham, MA). Homogenates were then subjected to two freeze/thaw cycles to further ensure cell lysis. Homogenates were clarified by centrifugation followed by sequential filtration through 0.45 and 0.2-µm syringe filters to remove cellular debris. Clarified samples (250 uL) were treated with 20 U RNase One (Promega, Madison, WI) and 30 U Turbo DNase (Ambion, Austin, TX) in 1x DNase buffer (Ambion) at 37°C for 90 minutes to degrade unprotected host and environmental nucleic acids. To ensure continuous DNase activity, 10 U of DNase was added to the sample every 30 minutes during the 90-minute incubation. Remaining nucleic acids were isolated using Trizol LS (Invitrogen, Carlsbad, CA) according to the manufacturer’s instructions. A final DNase treatment was performed to remove final traces of DNA from the RNA preparation.

Colostrum (approximately 4 mL) was manually collected from a SRLV seropositive ewe within the first 24 hours after giving birth. Colostrum was diluted 1:2 with cold phosphate-buffered saline and centrifuged at 800 x g for 15 minutes at 4°C. The cream layer was skimmed from the top and 250 µL of milk was treated with nucleases and RNA was extracted as described above.

RNA libraries were prepared as previously described ( Workman *et al.,* 2015; Workman *et al.,* 2017; Workman *et al.,* 2018). Briefly, 100 ng of total RNA were used as input material for Illumina TruSeq RNA Sample Preparation Kit (Illumina, San Diego, CA). Libraries were prepared as specified by the manufacturer’s protocol without the initial step of poly-A selection on oligo-dT beads. Libraries were sequenced on an Illumina MiSeq instrument with a 600-cycle kit (v3) to generate 2 × 300 bp paired-end reads. Index adapters were removed from raw sequence reads using cutadapt 1.9.1 ( Martin, 2011) or BBDuk 35.82 (Brian Bushnell within Geneious 11.1.4 ( Kearse *et al.,* 2012) (Biomatters, Auckland, New Zealand) and trimmed reads were screened against the UniVec_Core database to remove contaminating vector sequences. Overlapping paired-end reads were merged using BBMerge 8.9 (Bushnell within Geneious).

The remaining RNA was used to generate long-read sequencing libraries according to a modified RNA Iso-Seq with poly(A) tails added to the 3’ ends to allow cDNA synthesis of subgenomic fragments. The resulting cDNA was amplified, size fractionated and the largest fragments were used to make SMRTbell templates, which were sequenced on a Pacific Biosciences RSII instrument. SMRT Analysis was used to generate error corrected circular consensus sequences (CCS) from the raw reads and adapters and poly(A) tails were removed with BBDuk.

Reads greater than 1,000 nucleotides in length were assembled *de novo* with the Geneious assembler. All reads were then mapped to the *de novo* assembly, and the resulting consensus sequence was reported. Four strains lacked good quality long-read sequence data ( Table 1) so the MiSeq short reads were assembled using template-assisted assembly in Geneious following Workman *et al*. (2018) with accessions KY358787 and KY358788, respectively, used as subgroup 1 and 2 references. These two reference strains were included in this study.

To calculate total genome coverage for each sample, merged and unmerged paired-end reads plus long-read CCS’s were jointly mapped to the consensus genome using the Geneious mapper with 40% maximum allowable mismatches, a word length of 24, an index word length of 14, 10% allowable gaps and a maximum gap size of 50.

Up to 26 nucleotides on the 5’ ends did not fully resolve in genomes with only short-read sequencing available ( Table 1). Genomes were manually annotated based on alignments with full-length SRLV genomes available in GenBank.

### Phylogenetic analyses of full-length genomes

To augment the newly reported genome sequences, full-length SLRV and SLRV-like genomes were also downloaded from GenBank using the following query of the Nucleotide database on October 11, 2019: txid11660[ORGN] OR txid2169971[ORGN] OR txid11653[ORGN] OR txid11663[ORGN] AND ("8000"[SLEN] : "12000"[SLEN]). 79 unique genomes were aligned using MUSCLE 3.8.425 ( Edgar, 2004 in Geneious 11.1.5) and a neighbor net phylogenetic network was built using default settings in Splitstree5 ( Huson & Bryant, 2006).

### A2 Subgroup diagnostic SNP identification accounting for recombination

Population assignment of each genome generated in this study was performed in FineStructure version 4 and its companion program, ChromoPainter version 1 ( Lawson *et al.,* 2012). To generate the alignment used in FineStructure, MUSCLE was used followed by manual refinement. Gaps in the alignment were converted to presence/absence characters as-is with simple gap patterns reduced to a single character regardless of size. The recombination rate map used in FineStructure and Chromopainter was estimated using the LDhat 2.2a interval program ( McVean *et al.,* 2004). For LDhat, pre-computed likelihoods were generated using a population-scaled per-site mutation rate inferred from the data (0.07587), a grid size of 101 and a maximum population-scaled whole-genome recombination rate of 100. The variable rate estimation was run for 10 million iterations with the first half discarded as burn-in and a block penalty of 20. To avoid alignment edge inaccuracies, the final 10% of SNPs from the 3’ terminus were placed preceding the 5’ terminus and the first 10% of SNPs from the 5’ terminus were placed following the 3’ terminus, essentially simulating circular genomes. The recombination rate point estimates at these simulated edges were removed. The outputs of LDHat were population-scaled recombination rates ( *p*), which relate to the biochemical recombination rate ( *r*) according to the formula *p=2N _e_r* where *N _e_* is the effective population size. *N _e_* is difficult to estimate. Estimates for HIV, a related lentivirus which also produces chronic infections, vary by several orders of magnitude ( Pennings *et al.,* 2014). Computational estimates of *N _e_* in viruses also require time-series sampling data ( Rousseau *et al.,* 2017). Thus, *N _e_* was not estimated for this study and the Chromopainter recombination outputs were interpreted as being scaled by *2N _e_*. The FineStructure analysis was run using the linkage model, the variable recombination rate map estimated as described above, and specifications for haploid genomes. ChromoPainter detected shared ancestry by reconstructing each genome as a probabilistic mosaic of ‘chunks’ derived via recombination from all other input genomes (termed ‘all vs all painting’) and FineStructure assigned the genomes to populations based on the quantity and lengths of these shared genomic chunks. The following settings were changed from the default in FineStructure and/or ChromoPainter to ensure convergence of estimated parameters: ChromoPainter chunks-per-region parameter k=38, ChromoPainter samples s=10, Markov Chain Monte Carlo (MCMC) iterations=1e6 FineStructure tree finding maximization steps=1e6 and FineStructure independent MCMC runs=10. ChromoPainter iterations i=100 were run for estimating the global switch rate parameter and the global mutation rate. In FineStructure, Strain 201373037 was excluded from the estimation of the global switch rate parameter since it trended toward 0 and stalled the program. This indicated very closely related samples in the dataset (G. Hellenthal, personal communication). Inconsistency in assignment of individuals to populations was resolved by assigning all ambiguously assigned individuals to the largest of the potential populations.

To model subgroup-specific recombination, ChromoPainter version 2 was run in ‘donor-mode’ using the population assignments, global switch-rate parameter (0.168355), and population specific mutation rates output from the FineStructure analysis. These models were run for 500 iterations to ensure convergence of copy probability. In contrast to all vs all painting, donor specific painting assigns genomic chunks to recipient genomes based on donor populations comprised of multiple genomes. To increase genome-wide assignment probability of subgroup specific SNPs, consensus genomes with evidence of large recombination blocks were iteratively removed from each subgroup pool of donor genomes if average copy probability was increasing. This was done to eliminate the most obvious recombinants from the pools while retaining enough donor genomes to optimize the recombination model. The output donor subgroup-assignments for each SNP were used to identify subgroup-specific SNPs while accounting for recombination. Recombination rates have been estimated for several RNA retroviruses, and most estimates are in the range of 10 ^-3^ to 10 ^-5^ ( Tromas *et al.,* 2014). Thus, we also ran the diagnostic SNP identification model using a range of fixed recombination rates (10 ^-3^ to 10 ^-8^ Morgans-per-base pair) to confirm that diagnostic SNP count did not change when varying input recombination rate by several orders of magnitude.

### Dual infection inference using diagnostic SNPs

The subgroup-specific SNP content was quantified for each strain by extracting intra-host SNPs meeting default statistical restrictions (Maximum Variant P-value of 10 ^-6^, Minimum Strand-Bias P-value of 10 ^-5^ when exceeding 65% bias) relative to their final alignment in Geneious 11.1.4. The percentage of subgroup 1, subgroup 2, and ‘other’ SNPs at each diagnostic locus was calculated for each consensus genome. Subgroup partial dual infections were inferred as contiguous or nearly contiguous SNP blocks bearing both subgroup diagnostic alleles. For visualization relative to the consensus genome, these inferred partial dual infections were limited to genome blocks or scaffolds ≥50 nucleotides long where variants diagnostic for both subgroups co-occurred at a frequency of ≥5% and ≥2 reads. The 5% threshold was chosen as it was a conservative value accounting for sequencing error and mis-mapping when calling quasispecies SNPs. Multiple putative dual infection blocks were extended or scaffolded together when separated by <50 nucleotides and 1 diagnostic SNP. We characterize these as being caused by dual infection with unknown underlying viral haplotypes containing SNPs diagnostic to both subgroups at these regions as this is the most parsimonious explanation. However, quasispeciation in the absence of dual infection is an increasingly possible explanation as the numbers of adjacent subgroup diagnostic SNPs in the characterized regions decrease.

### Functional analyses and annotation

Once subgroup-specific SNPs were identified in the context of recombination, intra-host amino acid variation (functional quasispecies) at the subgroup diagnostic loci were identified by extracting variants from the alignments in Geneious 11.1.5 using the same statistical criteria applied to nucleotides and occurring at a frequency of ≥5%. Highly variable domains in *gag* and *env* previously shown to be important were analyzed in the context of subgroup assignment and host *TMEM154* diplotype. Additionally, SignalP-5.0 ( Nielsen, 2017) was used to predict the *env* signal peptide cleavage site.

## Results

### Genomes

**Figure 1.**
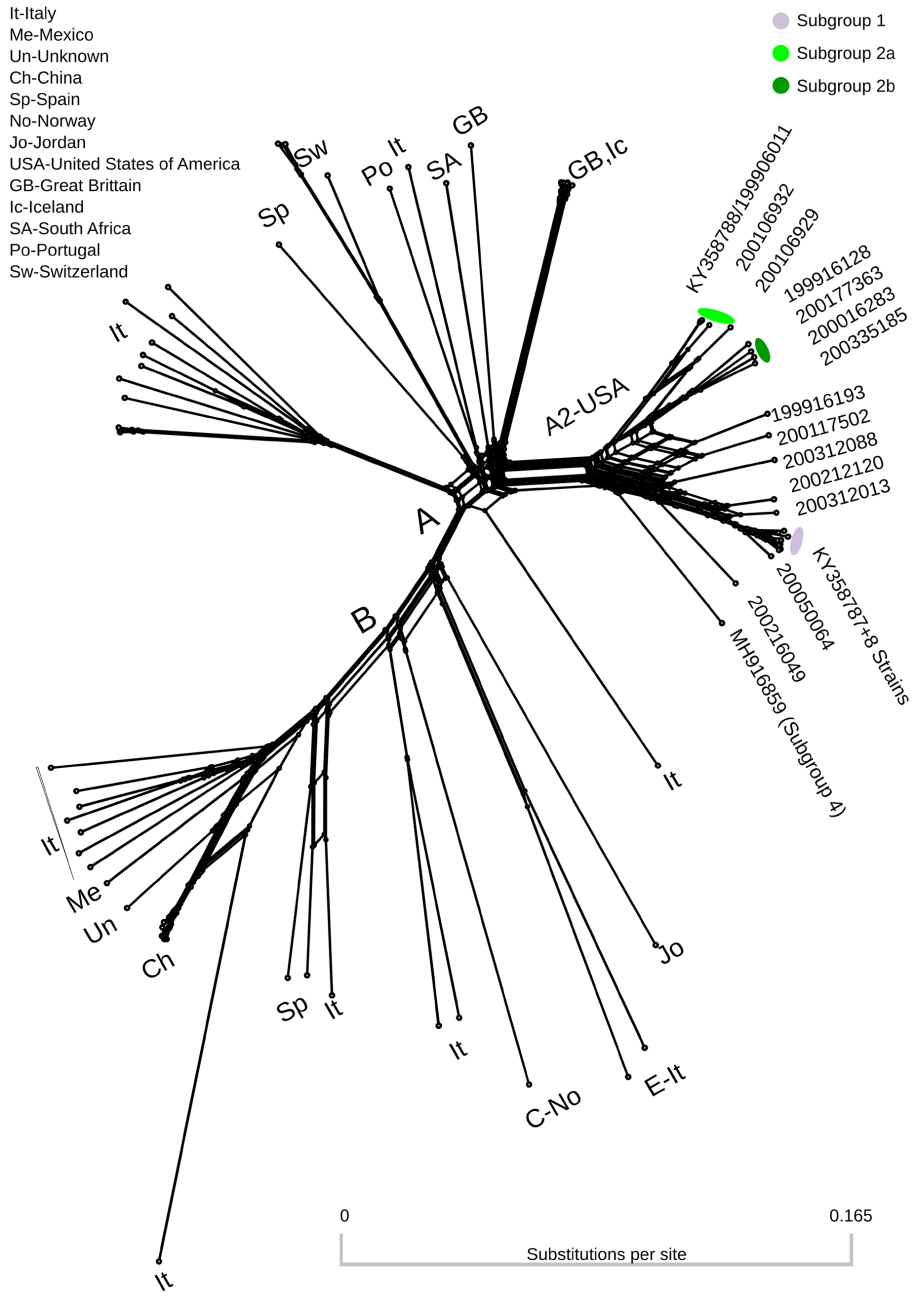
Splitstree neighbornet phylogenetic network of 79 SRLV genomes from genotypes A, B, C and E. Colors correspond to genomes assigned to subgroup specific pools of donor genomes in recombination analysis ( Table 1).

**Figure 2.**
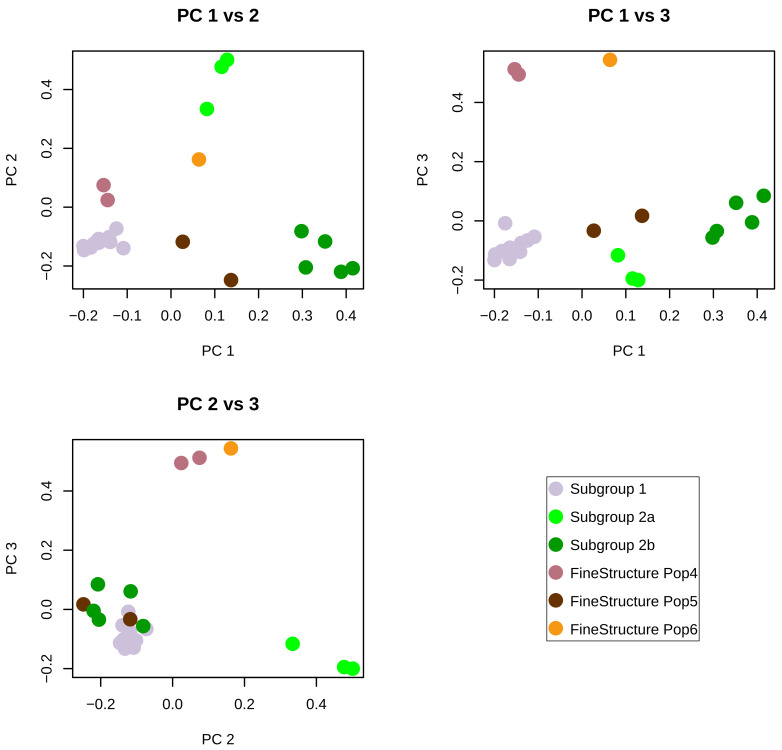
FineStructure population assignments of 23 SRLV subtype A2 genomes along the first three principal components. The first three principal components account respectively for 40, 18 and 11 percent of the variance in the data.

Coverage of the 23 genomes ranged from 52- to 2661-fold ( Table 1). Complete or near-complete genomes ranged from 9164 to 9215 nucleotides in length. The combined short read and long read consensus genome of strain 199906011 was slightly different from the long read only consensus (KY358788). The sites with differences had a high frequency of the minor allele in the quasispecies in the long read only consensus and so switched the identity of the minor allele in the combined short read and long read consensus. The combined short read and long read consensus of strain 200303013 was identical to the long read only consensus (KY358787). A phylogenetic network using full-length SRLV genomes was dominated by groups A and B ( Figure 1). Subtype A2 strains from the United States of America occupied a distinct cluster within the network. Additional clusters on the tree were generally represented by a single geographic region, with Italy representing the highest number of unique clusters. Recombination was evident in several clusters of the network including subtype A2. FineStructure analysis of the 23 genomes from this study identified six distinct populations across the consensus genomes based on all-vs-all genome painting ( Figure 2). FineStructure identified two distinct subgroup 2 populations identified in Figure 2 as subgroup 2a and subgroup 2b. Subgroup 2a is intermediate between subgroup1 and subgroup2b on principal component 1 ( Figure 2).

**Figure 3.**
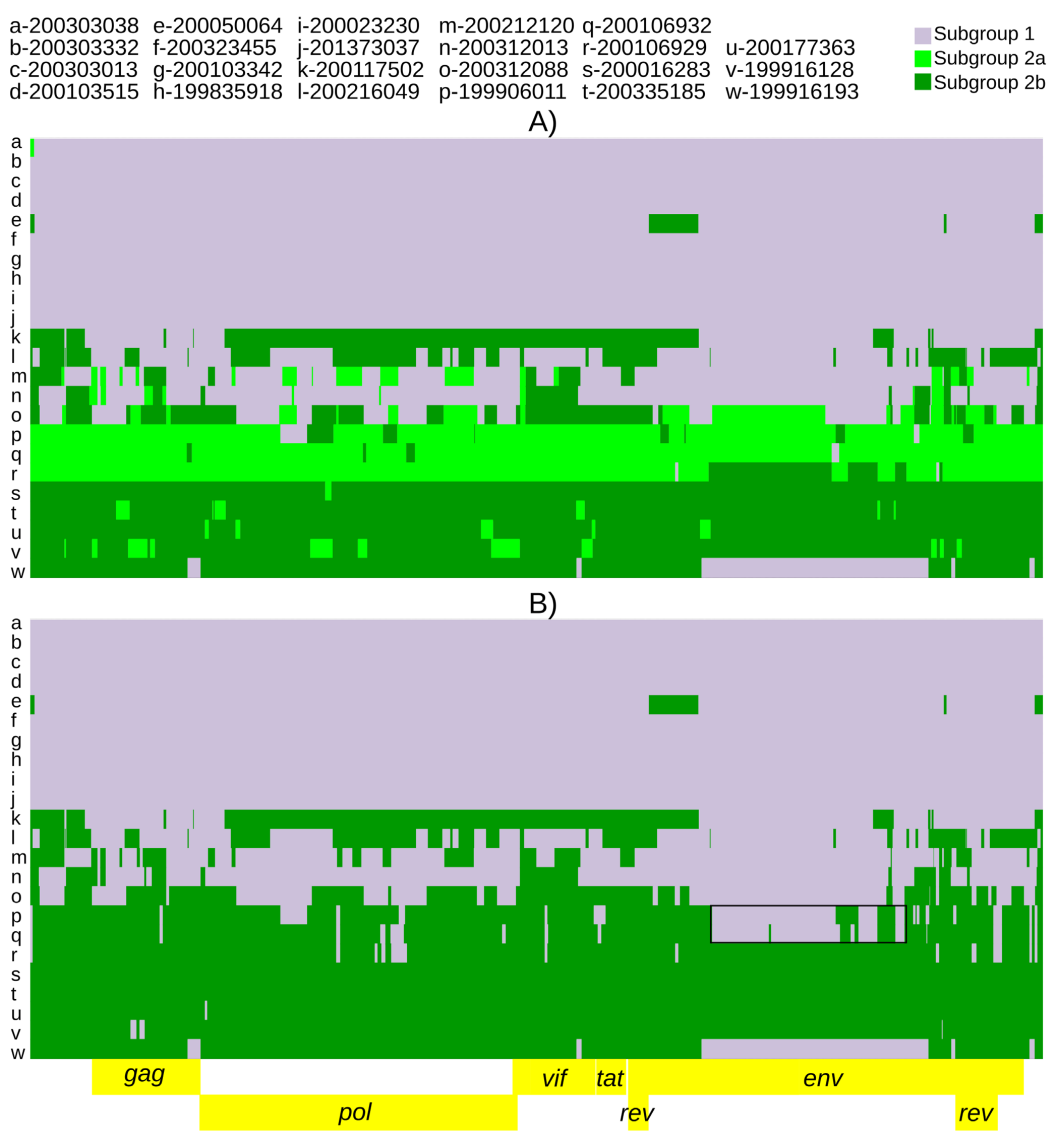
Twenty-three SRLV subtype A2 genomes ‘painted’ with recipient genomic ‘chunks’ derived from subgroup-specific donor genomes. The recombination models utilized were ( **A**) subgroup1 vs subgroup 2a vs subgroup 2b and ( **B**) subgroup 1 vs subgroup 2b. The black boxes highlight large subgroup 1 recombination blocks in subgroup 2a genomes.

### Recombination models

**Figure 4.**
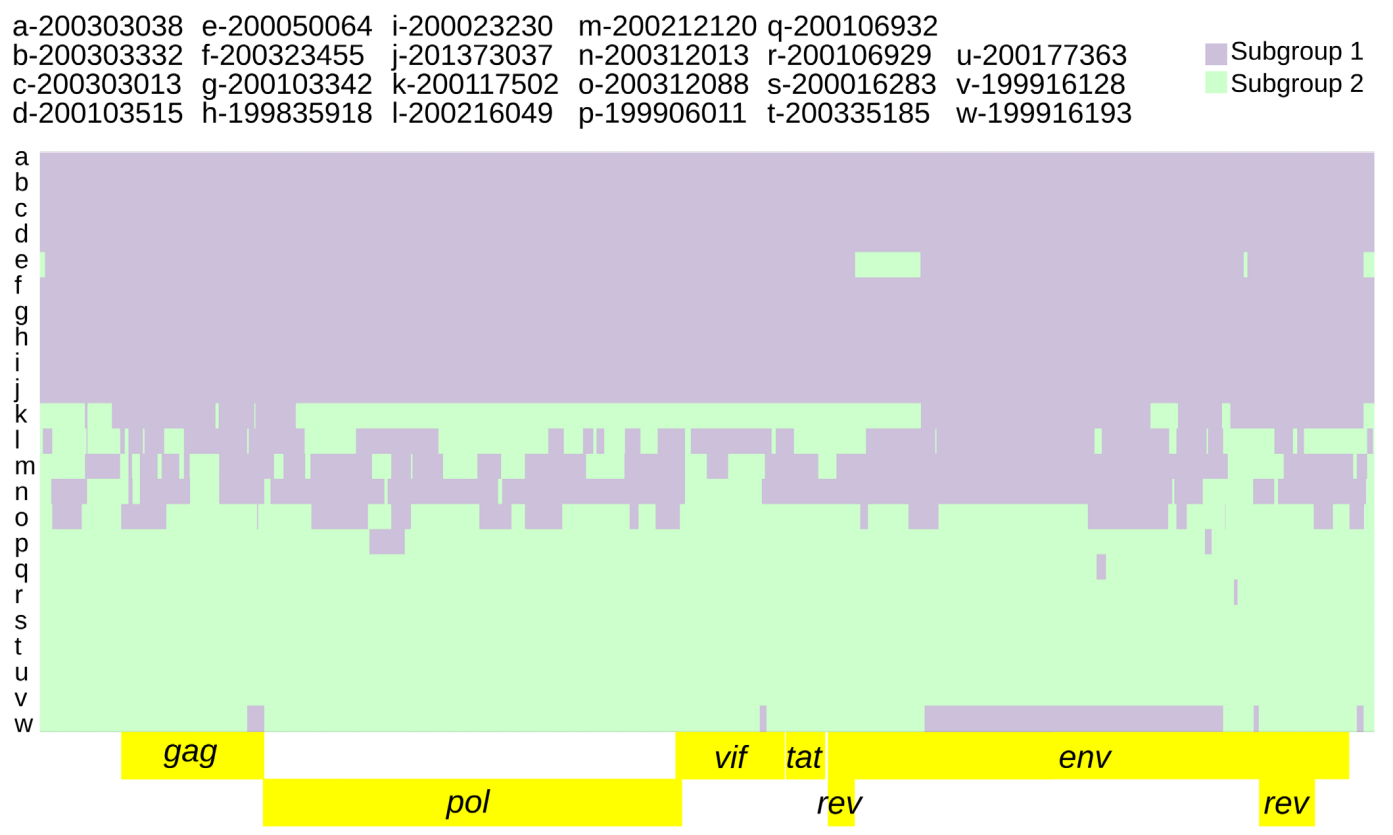
Twenty-three SRLV subtype A2 genomes ‘painted’ with recipient genomic ‘chunks’ derived from subgroup-specific donor genomes. The recombination model utilized was subgroup1 vs subgroup 2.

Due to the large number of FineStructure identified populations, several recombination models were run in ChromopainterV2. Five of the six identified populations had >1 consensus genome. The model with five potential populations of donor genomes indicated that the three most frequent populations (subgroup 1, subgroup 2a and subgroup 2b) accounted for >88% of the SNPs and were the majority donors to 22 genomes ( *Extended data*, Supplementary Figure 1 ( Dickey & Workman, 2020b)). The model was run with these three donor populations only ( Figure 3A). Due to the relative location of the four populations along principal component 1 ( Figure 2), the model was also run with subgroup 2b and subgroup 1 as the only two donor populations ( Figure 3B). This showed possible complex recombination blocks between subgroup 1 and subgroup 2b in subgroup 2a genomes ( Figure 3B). Subgroup 2a genomes also showed intermediate average pairwise percent divergences between subgroups 1 and 2b ( Table 3). All models showed many predicted recombination blocks spanning the consensus genomes ( Table 2, Figure 3 and *Extended data*, Supplementary Figure 1 ( Dickey & Workman, 2020b)). Finally, a recombination model was run with only 2 donor populations, subgroup 1 and subgroup 2 (as 2a+2b) ( Figure 4). This was done to identify and extract subgroup specific SNPs while accounting for recombination. Viral strains 200050064 and 199916128 were removed from the subgroup 1 and 2 donor pools respectively based on having the highest proportion of inter-subgroup recombination ( Table 2, Figure 3) and this improved the subgroup 1 vs subgroup 2 recombination model. Further removal of genomes as potential donors did not improve the model. The average number of alternate subgroup recombination blocks (Chromopainter’s chunkcount) was 2-fold higher in subgroup 2 genomes than subgroup 1 ( Table 2). Chromopainter’s chunklengths parameter averaged 3-fold higher in subgroup 2 and predicted population specific mutation rate averaged 3-fold higher in subgroup 2 consensus genomes than subgroup 1 ( Table 2).

**Table 2.** Select ChromopainterV2 calculated parameters for the subgroup 1 vs 2 recombination model ( Figure 4). Chromopainter’s ‘chunkcounts’ parameter is defined as the number of genomic chunks from a population of donor genomes, assigned to the recipient genome via recombination. ‘Chunklengths’ are the combined lengths (in centimorgans X 2 *N*_*e*_) of these chunks. Donor specific mutation rate is the amount of mismatching across the recipient chunklengths divided by the number of loci. Donor status is the population of donor genomes to which a consensus genome was assigned in the final subgroup 1 vs subgroup 2 recombination model ( Figure 4). To improve recombination models, genomes with high inter-subgroup recombination were iteratively removed from the populations if model quality (as judged by average copy probability) was increasing.

Recipient Genome	FineStructure Assigned Population	Donor Status	Subgroup 1 Chunk counts	Subgroup 2 Chunk counts	Subgroup 1 Chunk lengths	Subgroup 2 Chunk lengths	Donor specific mutation rate ^*^
200303038	Subgroup 1	Subgroup 1	52.4404	0	30218.2	0	0.0161771
200303332	Subgroup 1	Subgroup 1	51.8010	0	30218.2	0	0.0177648
200303013	Subgroup 1	Subgroup 1	60.0599	0	30218.2	0	0.0357224
200103515	Subgroup 1	Subgroup 1	60.6890	0	30218.2	0	0.0236503
200050064	Subgroup 1	Recipient only	48.2632	6.4329	28003.9	2214.3	
200323455	Subgroup 1	Subgroup 1	55.0174	0	30218.2	0	0.0196987
200103342	Subgroup 1	Subgroup 1	60.1737	0.0740	30207.5	10.7	0.0160127
199835918	Subgroup 1	Subgroup 1	61.1799	0	30218.2	0	0.0294231
200023230	Subgroup 1	Subgroup 1	62.7606	0	30218.2	0	0.0128684
201373037	Subgroup 1	Subgroup 1	46.9864	0	30218.2	0	0.0480409
200117502	Pop 5	Recipient only	25.5460	16.0997	15790.2	14428.0	
200216049	Pop 5	Recipient only	42.0507	43.0426	15384.2	14834.0	
200212120	Pop 4	Recipient only	43.3061	36.3817	18021.3	12196.9	
200312013	Pop 4	Recipient only	39.8241	22.9727	21460.6	8757.6	
200312088	Pop 6	Recipient only	27.2590	65.5737	8656.4	21561.9	
199906011	Subgroup 2a	Subgroup 2	2.8228	47.0027	582.9	29635.3	0.0628346
200106932	Subgroup 2a	Subgroup 2	1.0739	38.8866	469.4	29748.8	0.0525776
200106929	Subgroup 2a	Subgroup 2	1.4457	44.8749	339.5	29878.8	0.0684202
200016283	Subgroup 2b	Subgroup 2	0	63.3829	0	30218.2	0.0781928
200335185	Subgroup 2b	Subgroup 2	0	66.1020	0	30218.2	0.0929781
200177363	Subgroup 2b	Subgroup 2	0	70.5193	0	30218.2	0.0898418
199916128	Subgroup 2b	Subgroup 2	0	70.2713	0	30218.2	0.0938431
199916193	Subgroup 2b	Recipient only	6.1441	53.8583	5726.2	24492.0	-

*ChromopainterV2 mutationprobs parameter.

**Table 3.** Average pair-wise genetic distance within (on-diagonal) and among (off-diagonal) SLRV A2 subgroups characterized in this study.

	Subgroup 1	Subgroup 2a	Subgroup 2b
Subgroup 1	1.7%	13.7%	15.6%
Subgroup 2a	13.7%	5.1%	10.4%
Subgroup 2b	15.6%	10.4%	5.2%

Subgroup diagnostic SNP inference accounting for recombination between subgroup 1 and subgroup 2 (as FineStructure population 2a+2b) resulted in 413 diagnostic SNPs ( *Extended data*, Supplementary Table 1 ( Dickey & Workman, 2020a)). The frequency of alternate subgroup diagnostic alleles was 3-fold higher in subgroup 2 consensus genomes than subgroup 1 ( Table 4).

**Table 4.** Subgroup specific intra-host genetic variation (quasispecies) for 23 SRLV A2 consensus genomes. Donor status is the population of donor genomes to which a consensus genome was assigned in the final subgroup 1 vs subgroup 2 recombination model ( Figure 4).

Strain	Donor Status	Average % Subgroup 1 Across 413 Diagnostic Loci	Average % Subgroup 2 Across 413 Diagnostic Loci	Average % 'Other' Across 413 Diagnostic Loci	Genomic Regions Indicative of Dual Infection	Total Length of Genomic Regions Indicative of Dual Infection
200303038	Subgroup 1	99.79%	0.16%	0.05%	-	-
200303332	Subgroup 1	99.89%	0.02%	0.08%	-	-
200303013	Subgroup 1	99.55%	0.17%	0.28%	-	-
200103515	Subgroup 1	97.18%	1.75%	1.08%	-	-
200050064	Recipient only	91.70%	7.74%	0.56%	6	1427
200323455	Subgroup 1	99.25%	0.43%	0.32%	-	-
200103342	Subgroup 1	98.93%	0.73%	0.34%	-	-
199835918	Subgroup 1	98.89%	0.62%	0.49%	-	-
200023230	Subgroup 1	98.99%	0.50%	0.51%	-	-
201373037	Subgroup 1	98.78%	0.71%	0.51%	-	-
200117502	Recipient only	33.22%	66.24%	0.70%	9	3394
200216049	Recipient only	52.04%	47.62%	0.34%	13	3704
200212120	Recipient only	72.61%	25.48%	1.91%	11	4179
200312013	Recipient only	77.65%	21.69%	0.66%	8	1798
200312088	Recipient only	40.74%	58.20%	1.07%	14	2297
199906011	Subgroup 2	4.25%	95.12%	0.63%	1	158
200106932	Subgroup 2	1.00%	98.18%	0.82%	-	-
200106929	Subgroup 2	0.92%	98.49%	0.58%	-	-
200016283	Subgroup 2	0.93%	98.67%	0.40%	-	-
200335185	Subgroup 2	0.35%	99.27%	0.37%	-	-
200177363	Subgroup 2	1.62%	97.60%	0.79%	-	-
199916128	Subgroup 2	1.92%	96.40%	1.68%	2	241
199916193	Recipient only	19.47%	79.90%	0.62%	9	1896

Intra-host variation at subgroup-specific SNPs was analyzed to infer the presence of dual infections. The parameters specified for predicting subgroup dual infections resulted in 73 genomic regions indicative of dual infection across nine consensus genomes (range: 1–14, average: 8.2), averaging 261.6 nucleotides in length (range: 55–1482) and comprising 2%–45% of the genome ( *Extended data*, Supplementary Table 2 ( Dickey & Workman, 2020a), Figure 5).

**Figure 5.**
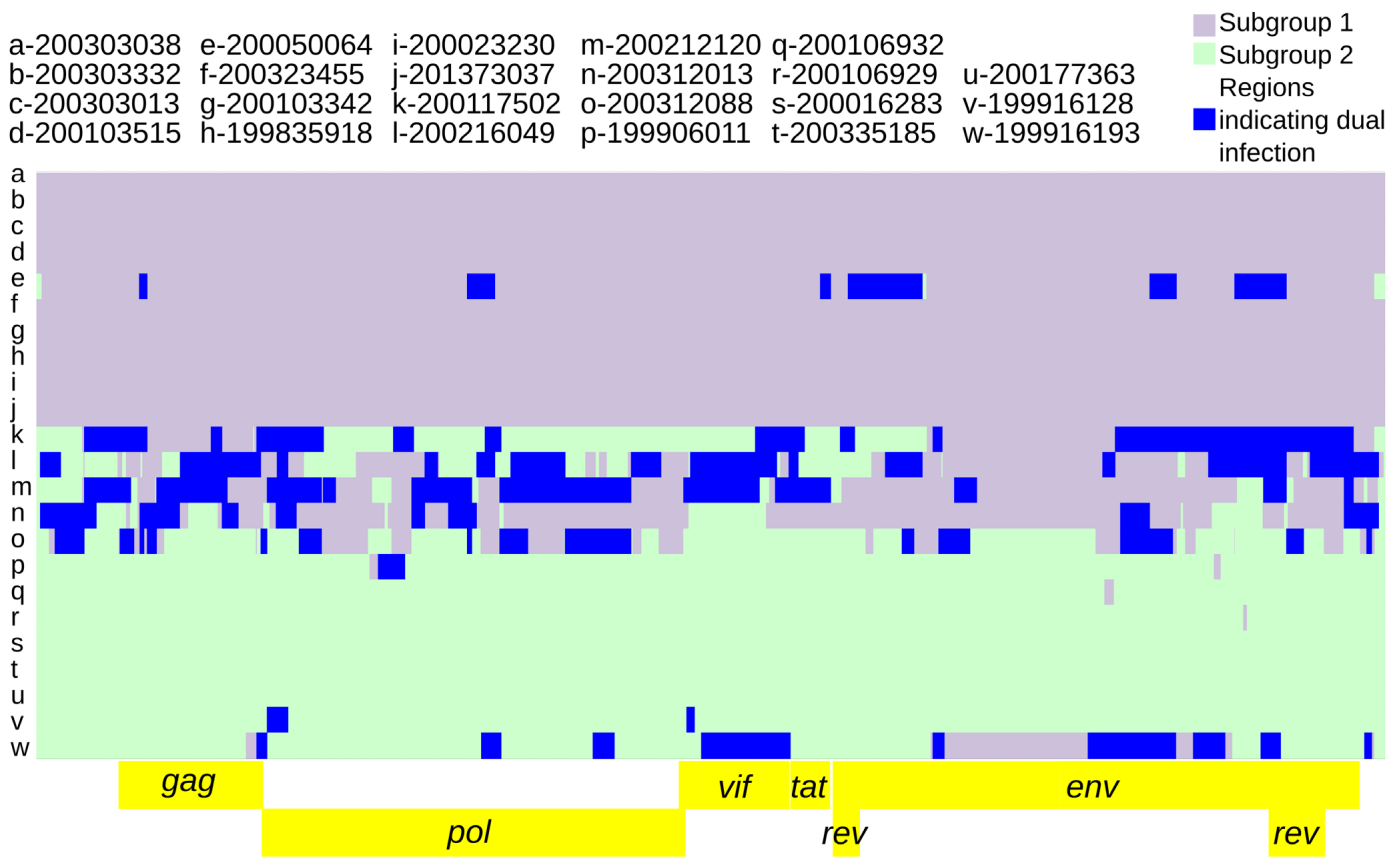
Twenty-three SRLV subtype A2 genomic regions indicative of dual infection. The background is the subgroup 1 vs subgroup 2 recombination model ( Figure 4). Genomic regions indicative of dual infection contained both subgroup diagnostic alleles at a frequency ≥5% for at least 2 consecutive diagnostic SNPs and 50 nucleotides.

### Functional variation

 Of the 413 subgroup diagnostic SNPs identified, 106 were non-synonymous ( *Extended data*, Supplementary Tables 1 and 3 ( Dickey & Workman, 2020a)). A2 subgroup 1 and 2 specific variants were identified in all viral genes with frequencies ranging from 2.2 to 4.3%. Sequence analysis of the immunodominant epitope in the *gag* gene revealed two adjacent SNPs that resulted in a single amino acid change distinguishing subgroups 1 and 2 ( *Extended data*, Supplementary Table 3 ( Dickey & Workman, 2020a)). Analysis of the *env* gene variable regions V1-V5 ( Valas *et al.,* 2000) found no subgroup specific SNP in variable regions V1 and V2, five subgroup specific variants each in V3 and V4, and one in V5 ( *Extended data*, Supplementary Table 3 ( Dickey & Workman, 2020a)). Six subgroup defining variants were identified in the predicted *env* signal peptide.

## Discussion

 This study provides full-length or near-full-length consensus genomes from 21 new SRLV subtype A2 strains used in determining the viral subgroup association with *TMEM154* E35K genotypes ( Heaton *et al.,* 2012; Sider *et al.,* 2013) in addition to the two subgroup representative strains from Workman *et al.,* 2017. These genomes were analyzed for recombination and population structure using a chromosome ‘painting’ model. Several genomes showed complex recombination patterns. Furthermore, this model was used to identify 413 subgroup-specific SNPs while accounting for recombination. This information was used to quantify intra-host genetic diversity at diagnostic SNPs and estimated nine animals were dually infected with viral recombinants such that they have diagnostic SNPs from both virus subgroups for portions of their genome. Lastly, we analyzed important functional domains in the virus genome in the context of virus subgroup and host *TMEM154* diplotypes focusing only on haplotypes 1, 2 and 3. 

The SRLV phylogenetic network contained subtype A2 as a distinct cluster ( Figure 1). Several genomes in this cluster are connected by many nodes indicating inter-subgroup recombination. The FineStructure analysis identified two distinct subgroup 2 ‘populations’ of consensus genomes. These have been provisionally designated subgroup 2a and subgroup 2b. Subgroup 2a is intermediate between subgroup 1 and subgroup 2b both in terms of position along principal component 1 ( Figure 2) and in terms of genetic distance ( Table 3). The subgroup 2 genome reported by Workman *et al.,* 2017 (viral strain 199906011, GenBank KY358788.1) belongs to subgroup 2a ( Table 1). This is a case where it is difficult to distinguish, with certainty, the recombinant from the second viral donor, however there is some evidence that subgroup 2b may be a ‘purer’ representative of subgroup 2. Even when subgroup 2a genomes were included among the subgroup 2 donors, the only consensus genomes that resolved unambiguously as subgroup 2 were the four subgroup 2b strains ( Table 2). In contrast, subgroup 1 was more clearly delimited by the recombination model. Of the nine subgroup 1 donor genomes, only 200103342 did not resolve unambiguously as subgroup 1 ( Table 2).

Subgroup 2 was more genetically diverse than subgroup 1 based on its higher mutation rates and increased recombination ( Figure 4, Table 2). Subgroup 2 also had higher intra-host genetic diversity based on the dual infection analysis ( Table 4). The consensus genomes with the highest intra-host diversity ( Figure 5, Table 4 and *Extended data*, Supplementary Table 2 ( Dickey & Workman, 2020a)) and highest recombination block count ( Figure 4, Table 2) did not conform to a good concept of ‘population’ ( *Extended data*, Supplementary Figure 1 ( Dickey & Workman, 2020b)) despite being identified as populations by FineStructure analysis ( Figure 2). But these genomes could be parsimoniously modeled as complex recombinants of subgroups 1 and 2 ( Figure 4). We argue that more than one representative genome is required to properly distinguish subgroups from recombinant forms of subgroups 1 and 2. The subgroup 1 vs subgroup 2 recombination model used to identify diagnostic SNPs was informed by both recombination rate variation across the genome as well as differing mutation rates between subgroups. Models run with a constant recombination rate across the genome identified a slightly higher number (421) of diagnostic SNPs (see *Methods*). The dual infection analysis identified multiple genomic regions featuring modest frequencies of both subgroup diagnostic alleles ( *Extended data*, Supplementary Table 2 ( Dickey & Workman, 2020a)). These were identified in the most recombinant consensus genomes ( Figure 4). Because the dual infection regions did not span the entire genome, the underlying haplotypes were most likely recombinant as opposed to ‘pure’ subgroup sequences.

The relatively small number of strains characterized in this study and a paucity of geographic variability biased our results and limited our ability to make recombination-based inference. While the subset of samples from the original *TMEM154*/A2 association studies chosen was a good starting place for modeling recombination, the addition of larger numbers of geographically diverse SRLV A2 genomes should improve the recombination model(s) substantially due to a larger pool of potential recombinant and parental genomes ( Yahara *et al.,* 2019). There are presently 79 unique full length SRLV genomes available with more than half of these published since 2019 ( Colitti *et al.,* 2019; present work) so the time has probably come to recharacterize SRLV diversity at the whole-genome level, expanding the current classification beyond partial *gag*/ *pol* sequence. A revised classification system will better facilitate outbreak tracing and identification of recombinants circulating beyond their local flocks. Such circulating recombinant forms (CRFs) have been extensively characterized for HIV ( Carr *et al.,* 1999; Leitner *et al.,* 2005) providing a possible model and framework for the SRLV research community to adapt. However, the current CRF framework for HIV utilizes consensus genomes so an accounting of the underlying haplotypes (quasispecies) contributing to these consensuses would benefit the genomic characterization of both lentiviruses.

Fras *et al.,* 2013 have suggested that dual infection of small ruminant lentiviruses may be common, understudied, and underreported. Our results and those of Sider *et al.,* 2013 confirm that dual infection is common though none of our samples showed evidence of having been dually infected by pure subgroup 1 and 2 representatives. Hopefully, declining sequencing costs and increased availability of whole genome sequencing will foster greater reporting of this phenomenon. Our results also conform to those of Sider *et al.,* 2013 including the two subgroups identified and the existence of recombination. These results extend those of Sider *et al.,* 2013 from partial *gag*/ *env* to the complete genome while accounting for recombination. Recombination is also clearly delimited by the ChromoPainter models. The largest recombination blocks were also predicted by the software program, RDP ( Martin *et al.,* 2015) (data not shown), which also identified the largest 1+2b=2a recombination block spanning the middle portion of *env* ( Figure 3B, individuals p and q). While our results extend the existence of two subgroups across the entire SRLV genome, subgroup 2 has additional population sub-structuring ( Figure 2, Figure 3B). Subgroup 2a may represent a somewhat stable locally circulating recombinant of subgroup 1 and 2b ( Figure 3). The genomes identified as 2a were found exclusively in *TMEM154* 2,2 and 2,3 diplotype animals ( Table 1). Additionally, most strains with genomic regions indicative of dual infection were from *TMEM154* susceptible 1,2 and 1,3 heterozygotes, i.e. animals with both an E and K at position 35. This suggests that animals that are E35K heterozygous due to TMEM *154* 1,2 and 1,3 diplotypes may facilitate recombination between subgroups 1 and 2.

Interestingly, two subtype specific functional variants were found in a region of the *env* gene variable region 4 (V4) which was recently identified to contain “signature patterns” associated with different clinical status in sheep and goats ( Mendez *et al.,* 2020). This region of V4 also contains targets of neutralizing antibodies and is predicted to play a role in virus entry ( Skraban *et al.,* 1999). Multiple amino acid changes were also observed in the N-terminus of *env*. None of the amino acids were predicted to change the *env* signal peptide cleavage site; however, it would be interesting to know if the five subgroup-specific amino acids affect post-translational modifications such as cleavage timing, folding, or glycosylation, phenomena documented to affect HIV fitness ( Asmal *et al.,* 2011; Snapp *et al.,* 2017; Upadhyay *et al.,* 2018). As more genomes are sequenced, and we learn more about the function of *TMEM154* in the context of the virus lifecycle, it will be interesting to see which, if any, of these viral sequences are biologically responsible for *TMEM154* associations.

## Data availability

### Underlying data

NCBI sequence accessions are provided in Table 1.

### Extended data

Figshare: Supplemental Tables. https://doi.org/10.6084/m9.figshare.13109984 ( Dickey & Workman, 2020a).

This project contains the following extended data:

- Supplemental Table 1. Four hundred and thirteen diagnostic SNPs distinguishing SRLV A2 subgroups.
- Supplemental Table 2: Seventy-three genomic regions indicative of dual subgroup infection among 9 SRLV A2 genomes.
- Supplemental Table 3: Within-host amino acid variability (functional viral quasispecies) at 106 subgroup diagnostic loci. Colors according to subgroup 1 vs subgroup 2 recombination model ( Figure 4) can be seen in the downloaded file.

Figshare: Supplemental Figure 1. https://doi.org/10.6084/m9.figshare.13109909 ( Dickey & Workman, 2020b).

This file contains 23 SRLV subtype A2 genomes ‘painted’ with recipient genomic ‘chunks’ derived from populations of donor genomes. The five donor genome populations in the recombination model were determined using FineStructure.

Extended data are available under the terms of the Creative Commons Attribution 4.0 International license (CC-BY 4.0).
