# Supplementary material for: Classification of small ruminant lentivirus subtype A2, subgroups 1 and 2 based on whole genome comparisons and complex recombination patterns
Source: F1000Res. 2021 Jul 26;9:1449. Originally published 2020 Dec 11. [Version 2] doi: 10.12688/f1000research.27898.2 (PMC8749911; doi:10.12688/f1000research.27898.2)
Supplement: Peer review history

# Peer review: Reviewer response for version 2

**DOI:** 10.5256/f1000research.58784.r115360

**Published:** 2022-01-10

**Authors:** Jorge Hugo Calvo

**Affiliations:** 1 Centro de Investigación y Tecnología Agroalimentaria de Aragón, Instituto Agroalimentario de Aragón (IA2), CITA-Zaragoza University, Zaragoza, Spain; 2 ARAID, Zaragoza, Spain

In this manuscript, the authors assembled the full-length viral genome sequence of 23 subtype A2 SRLV strains using long-read sequencing technology, and try to identify recombination patterns. The authors compare the 79 unique full-length SRLV genomes. I am reviewing the version 2 of this manuscript that has improved the previous version. The article is well-written, the results of the study are well presented, well-supported, and certainly add to the current knowledge of the topic. Authors could include the prevalence of the different  SRLV subtypes in the breed and the geographical area,  if it is known. The authors state that ewes heterozygous for E35K (TMEM154 1,2 and 1,3 diplotypes) may facilitate recombination between subgroups 1 and 2, but they only have 23 full length viral genomes, and this should be confirmed using a bigger experimental design.

 In my opinion, the manuscript is acceptable for indexing.

Is the work clearly and accurately presented and does it cite the current literature?

Yes

If applicable, is the statistical analysis and its interpretation appropriate?

Yes

Are all the source data underlying the results available to ensure full reproducibility?

Yes

Is the study design appropriate and is the work technically sound?

Yes

Are the conclusions drawn adequately supported by the results?

Yes

Are sufficient details of methods and analysis provided to allow replication by others?

Yes

Reviewer Expertise:

Research focused in the application of genomic tools in animal science, mainly in ruminants, including animal breeding, nutrigenomics and nutrigenetics, and also in animal health.

I confirm that I have read this submission and believe that I have an appropriate level of expertise to confirm that it is of an acceptable scientific standard.

# Peer review: Reviewer response for version 2

**DOI:** 10.5256/f1000research.58784.r90371

**Published:** 2021-07-27

**Authors:** Monika Olech

**Affiliations:** 1 Department of Biochemistry, National Veterinary Research Institute, Puławy, Poland

The authors have addressed my concerns. Manuscript is now acceptable for indexing in this revised version.

Is the work clearly and accurately presented and does it cite the current literature?

Partly

If applicable, is the statistical analysis and its interpretation appropriate?

I cannot comment. A qualified statistician is required.

Are all the source data underlying the results available to ensure full reproducibility?

Yes

Is the study design appropriate and is the work technically sound?

Yes

Are the conclusions drawn adequately supported by the results?

Partly

Are sufficient details of methods and analysis provided to allow replication by others?

Yes

Reviewer Expertise:

Genetic and anitigenic characterization of Small Ruminant Lentiviruses (SRLV) in sheep, goats and wildlife ruminants (molecular analysis, phylogeny, cross species infection, genetic recombination)

I confirm that I have read this submission and believe that I have an appropriate level of expertise to confirm that it is of an acceptable scientific standard.

# Peer review: Reviewer response for version 1

**DOI:** 10.5256/f1000research.30850.r83037

**Published:** 2021-04-28

**Authors:** Carlos Loncoman

**Affiliations:** 1 Applied Biochemistry Laboratory, Institute of Pharmacology and Morphophysiology, Faculty of Veterinary Sciences, Universidad Austral de Chile, Valdivia, Chile

In this manuscript the authors assembled full length viral genomes and then analyzed specific segments in order to identify recombination patterns by using chromosome painting model.

 General comments:

 It’s not clear in the introduction whether this chromosome painting model is used for recombination detection in any other virus? Perhaps any other RNA virus at all? I would highly appreciate if the authors could expand their introduction about this.

 I would suggest the authors to include RDP software in methods and at least 1 figure in the results to see where the recombination events take place within the genome and compare these results with the chromosome painting one. Currently, these results are as not shown so it’s not very clear whether these results are complementary.

 My major concern with the recombination events that the authors describe in the manuscript are related with the differentiation between mutation and recombination. They mention in the introduction that RNA viruses have more mutations due to the low fidelity of the reverse transcriptase how did they differentiate these events from true recombination events?

 Specific comments:

 Abstract - Methods – The goal. Could the author explain the method rather than the goal?

 In the first paragraph would be good to read 1 or 2 lines about the importance or impact of this pathogen over sheep population not just the disease that cause the virus. Perhaps include incidence or any statistics about the disease that cause the virus. This would help to put this research in context.

 As the genome contains more than one non-coding long terminal repeat regions. I wonder how did the authors manage to sequence that specific part as I know they could be problematic to determine sequence identity. Are these regions inverted?

 Why the authors did not amplify the virus previous to sequencing? Is there any cell culture that could be used for this purposes? Could this help with the four strains that had low quality long read sequence?

 RDP software is mentioned in the discussion but not in the methods.

 The split tree software has a tool to measure recombination called PHI test. Is there a reason why the authors did not include this in the manuscript?

 What does PC stands for in Figure 2?

Is the work clearly and accurately presented and does it cite the current literature?

Yes

If applicable, is the statistical analysis and its interpretation appropriate?

I cannot comment. A qualified statistician is required.

Are all the source data underlying the results available to ensure full reproducibility?

Partly

Is the study design appropriate and is the work technically sound?

Yes

Are the conclusions drawn adequately supported by the results?

Yes

Are sufficient details of methods and analysis provided to allow replication by others?

Partly

Reviewer Expertise:

Virology; recombination; herpesvirus

I confirm that I have read this submission and believe that I have an appropriate level of expertise to confirm that it is of an acceptable scientific standard, however I have significant reservations, as outlined above.

# Response

**Published:** 2021-07-16

**Authors:** Aaron Dickey

**Affiliations:** USDA, USA

General comments:

1. It’s not clear in the introduction whether this chromosome painting model is used for recombination detection in any other virus? Perhaps any other RNA virus at all? I would highly appreciate if the authors could expand their introduction about this. Response: The recombination modeling program utilized in these analyses has been previously used to analyze recombination in *Helicobacter pylori* prophages (Yahara et al., 2019) and Herpes Simplex Virus (Forni et al., 2020). This information has been added to the methods. The Yahara et al. reference may be most similar in initial approach. Ours may be the first use of ChromoPainter version 2 ‘donor mode’ to extract subgroup-diagnostic variants in viruses while accounting for recombination.
2. I would suggest the authors to include RDP software in methods and at least 1 figure in the results to see where the recombination events take place within the genome and compare these results with the chromosome painting one. Currently, these results are as not shown so it’s not very clear whether these results are complementary. Response: We agree with the reviewer that our use of RDP belongs in the methods. We have attempted to do so in a way that does not distract from the primary focus of the manuscript, which was to identify subgroup-diagnostic variants, rather than recombination events. RDP was only used in this work to confirm a single putative event: the possible introgression of a large chunk of subgroup 1 sequence into a subgroup 2b background to create subgroup 2a (black box in Figure 3b). This event (approximate size, genomic position, and ChromoPainter assigned subgroup of RDP identified putative parental strains) was confirmed by RDP. This information has been added to the results and the details of the RDP event are provided in Extended data, Supplemental Table 2(Dickey & Workman, 2020a).
3. My major concern with the recombination events that the authors describe in the manuscript are related with the differentiation between mutation and recombination. They mention in the introduction that RNA viruses have more mutations due to the low fidelity of the reverse transcriptase how did they differentiate these events from true recombination events? Response: All recombination analysis programs claim to account for mutation as this is a fundamental issue. ChromoPainter initializes at the mutation rate of Li and Stephens 2003, but we estimated it in our analyses by using the -iM flag according to program developer recommendations. Interestingly, ChromoPainter’s mutation rate estimates are 3-fold higher for subgroup 2 genomes compared to subgroup 1 (Table 2). Population specific mutation rate estimates were obtained by averaging the estimated rates from the fineSTRUCTURE run among members of the assigned population and incorporated into our increasingly refined recombination models using the -m flag. We have added this information and the Li and Stephens reference to the manuscript. Our subgroup-specific-colored blocks in figures 3-5 are best interpreted as regions of sequence assigned by the recombination model to those subgroups, not as events-per-se. We believe regions alternating back-and-forth between subgroups over short genomic spans (e.g. strains labeled k-r in figures 3-5), are sometimes best interpreted as having arisen from multiple recombination events over time. We have attempted to minimize the use of the term ‘event’ in the manuscript in service of this paradigm.

Li N, Stephens M (2003) Modeling linkage disequilibrium and identifying recombination hotspots using single-nucleotide polymorphism data. Genetics 165: 2213–2233

 Specific comments:

1. Abstract - Methods – The goal. Could the author explain the method rather than the goal? Response: The methods section now reads: Methods: Sequence variation within and among full-length assemblies of SRLV subtype A2 subgroups 1 and 2 was analyzed to identify genome-scale recombination patterns.
2. In the first paragraph would be good to read 1 or 2 lines about the importance or impact of this pathogen over sheep population not just the disease that cause the virus. Perhaps include incidence or any statistics about the disease that cause the virus. This would help to put this research in context. Response: This information has been added to the first paragraph of the introduction.
3. As the genome contains more than one non-coding long terminal repeat regions. I wonder how did the authors manage to sequence that specific part as I know they could be problematic to determine sequence identity. Are these regions inverted? Response: We used PacBio long-read technology to help resolve the ends of the viral genome.  We report some genomes as ‘near-complete’ because we did not obtain the complete LTR sequence for all genomes.
4. Why did the authors not amplify the virus previous to sequencing? Is there any cell culture that could be used for this purposes? Could this help with the four strains that had low quality long read sequence? Response: It is true that the virus can be isolated in culture prior to sequencing; however, there were no commercially available cell lines for the isolation of SRLV at the time we initiated this project. While we could have developed appropriate cell lines, we chose not to isolate the virus first, as this can lead to the selection of a subpopulation of the infecting virus quasispecies. For these reasons, we sequenced the virus directly from the infected tissues.
5. RDP software is mentioned in the discussion but not in the methods. Response: Please see the response to General comment 2.
6. The split tree software has a tool to measure recombination called PHI test. Is there a reason why the authors did not include this in the manuscript? Response: SplitsTree5 does not have this analysis option, though we also remember it from SplitsTree4. We ran a PHI-test in RDP5 and now report it in the results.
7. What does PC stand for in Figure 2? Response: Principal Component (PC), is now incorporated into the legend of Figure 2.

# Peer review: Reviewer response for version 1

**DOI:** 10.5256/f1000research.30850.r77216

**Published:** 2021-02-08

**Authors:** Monika Olech

**Affiliations:** 1 Department of Biochemistry, National Veterinary Research Institute, Puławy, Poland

In this article, the authors focused mainly on the analysis of recombination of 23 SRLV strains belonging to subtype A2. In this paper a lot of different analyses were performed, but the results and figures are described very poorly. This makes the article very hard to read. For example on Fig. 1 I see only 15 isolates not 23. Lack is information on accesion numbers or isolate names of sequences from GenBank present on the tree. Please indicate to which genotypes they belong.

 In Figure 2, there are three images that should be labeled as A, B and C and their description should be more detailed (in figure and in results). In the third picture I see three populations not six.

 I don't understand why all 23 sequences were used in the recombination analysis. The authors write that only subgroubs 2a, 2b and 1 was used in the reombination models but some genomes not belonged to these subgroups (Fine structure Pop4, 5 and 6, Figure2).

 In the discussion the results of the study are repeated.

 There are no analyses regarding the occurrence of TMEM154E35K genotypes and susceptibility/resistance of sheep infected with and SRLV A2 subgroups 1 and 2. Did the research confirm previous studies described by Sider *et al.*, 2013?

Is the work clearly and accurately presented and does it cite the current literature?

Partly

If applicable, is the statistical analysis and its interpretation appropriate?

I cannot comment. A qualified statistician is required.

Are all the source data underlying the results available to ensure full reproducibility?

Yes

Is the study design appropriate and is the work technically sound?

Yes

Are the conclusions drawn adequately supported by the results?

Partly

Are sufficient details of methods and analysis provided to allow replication by others?

Yes

Reviewer Expertise:

Genetic and anitigenic characterization of Small Ruminant Lentiviruses (SRLV) in sheep, goats and wildlife ruminants (molecular analysis, phylogeny, cross species infection, genetic recombination)

I confirm that I have read this submission and believe that I have an appropriate level of expertise to confirm that it is of an acceptable scientific standard, however I have significant reservations, as outlined above.

# Response

**Published:** 2021-07-16

**Authors:** Aaron Dickey

**Affiliations:** USDA, USA

1. In this article, the authors focused mainly on the analysis of recombination of 23 SRLV strains belonging to subtype A2. In this paper a lot of different analyses were performed, but the results and figures are described very poorly. This makes the article very hard to read. For example on Fig. 1 I see only 15 isolates not 23.  Response: Figure 1 is information dense, particularly the very similar subgroup 1 genomes denoted by the lavender-colored oval. However, we agree it is beneficial to list all genomes, so they are now listed in clockwise order in the new Supplemental Table 1.
2. Lacking is information on accession numbers or isolate names of sequences from GenBank present on the tree.  Response: An added benefit to the new supplemental table (see previous comment) is space to put the GenBank accession numbers of all strains in Figure 1. Genbank accession number, isolate name, county of origin, and virus genotype are now listed in Supplemental Table 1.
3. Please indicate to which genotypes they belong. Response: This information is made clearer in the figure by curly brackets, additional annotations, and clarification in the legend.
4. In Figure 2, there are three images that should be labeled as A, B and C and their description should be more detailed (in figure and in results). In the third picture I see three populations not six. Response: The figures sections have been relabeled A, B and C and additional details are provided in the results. We are not certain but think the reviewer may be referring to 3 clusters of strains, not six, since principal components 2 and 3 (PC2 and PC3) do not strongly discriminate subgroups 1 and 2b.  We do not find this problematic since the first principal component strongly discriminates these two populations.
5. I don't understand why all 23 sequences were used in the recombination analysis. The authors write that only subgroups 2a, 2b and 1 was used in the recombination models but some genomes not belonged to these subgroups (Fine structure Pop4, 5 and 6, Figure2). Response: Our initial recombination donor model had five donor populations and all 23 genomes as possible recipients (Supplemental Figure 1). fineSTRUCTURE population 6 was excluded from this analysis on the grounds that singletons could not comprise a population for this type of analysis. The result suggests fineSTRUCTURE populations 4 and 5 are probably not populations either, justifying our continued refinement of recombination models to those with fewer donor populations, which better characterize the subgroups for which there is sufficient evidence, and recombination patterns among them. Our explanation of this is that fineSTRUCTURE assigned ‘population’ status to groups of genomes that would be better described as complex recombinants of already defined subgroups, rather than true populations. Over-splitting of these types of datasets into too many ‘populations’ is a known problem. See: Falush D, van Dorp L, Lawson D, 2016 A tutorial on how (not) to over-interpret STRUCTURE/ADMIXTURE bar plots. bioRxiv 066431; 10.1101/066431. While, fewer potential donor genomes were used in the refined recombination models (Figures 3 and 4), all genomes were used as recipients, allowing us to characterize recombination.
6. In the discussion the results of the study are repeated. Response: We believed this was beneficial to organizing points of discussion.
7. There are no analyses regarding the occurrence of TMEM154E35K genotypes and susceptibility/resistance of sheep infected with and SRLV A2 subgroups 1 and 2. Did the research confirm previous studies described by Sider et al., 2013? Response: We generated complete genome sequences from a subset of genomes characterized in the Sider et al., 2013 paper.  The goal was not to confirm or dispute the association, but rather, to generate complete genomes and develop a nucleotide polymorphism typing system for the two major subgroups while accounting for recombination. More complete SRLV sequences are required to identify which viral elements correlate with host adaptation.
